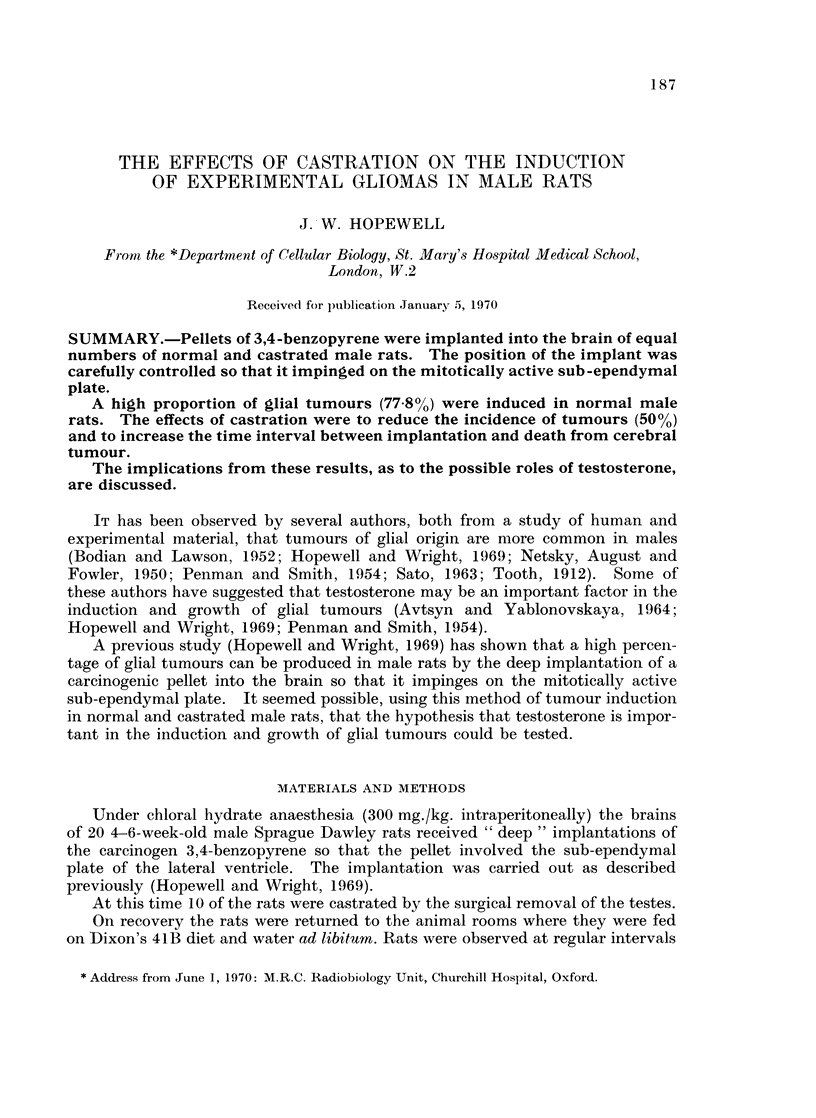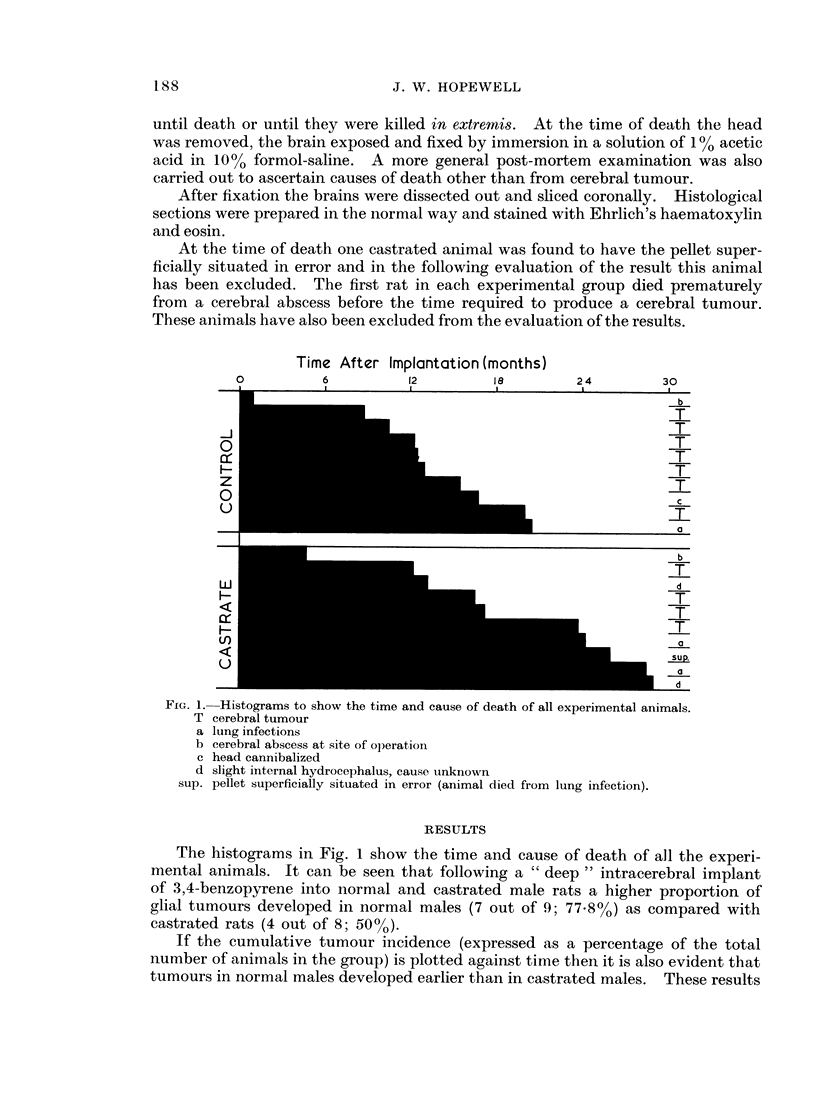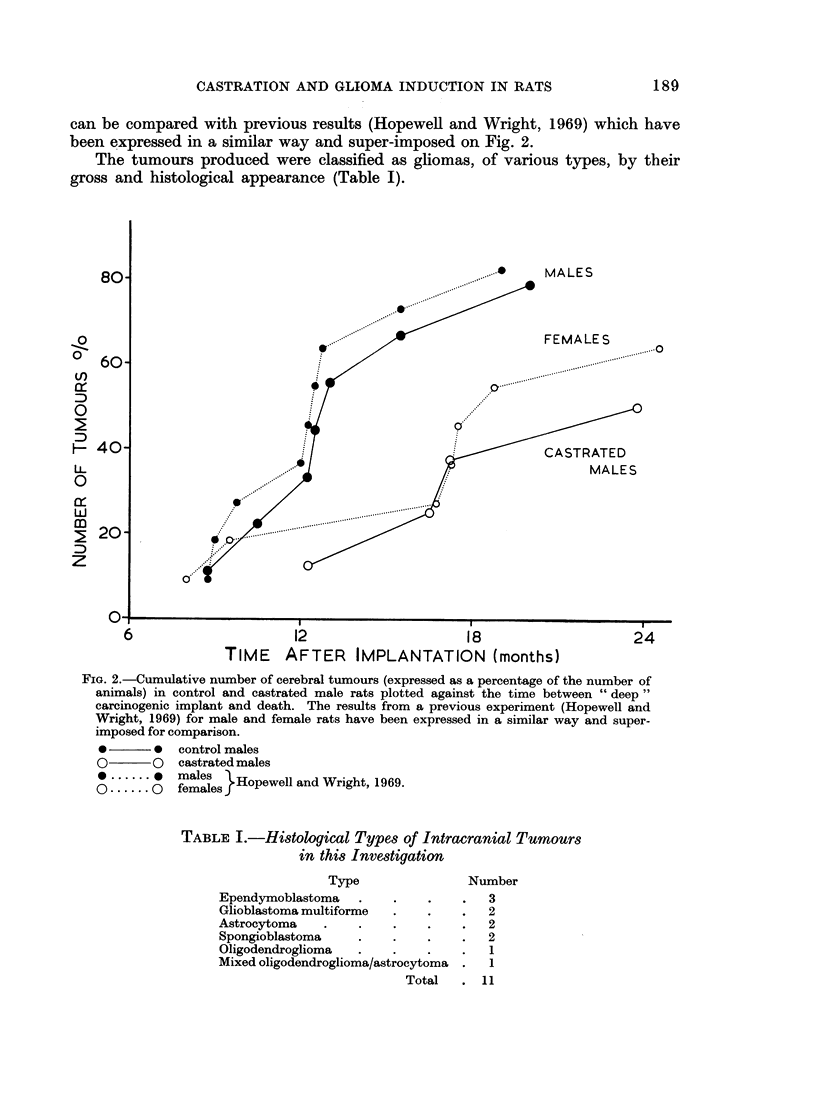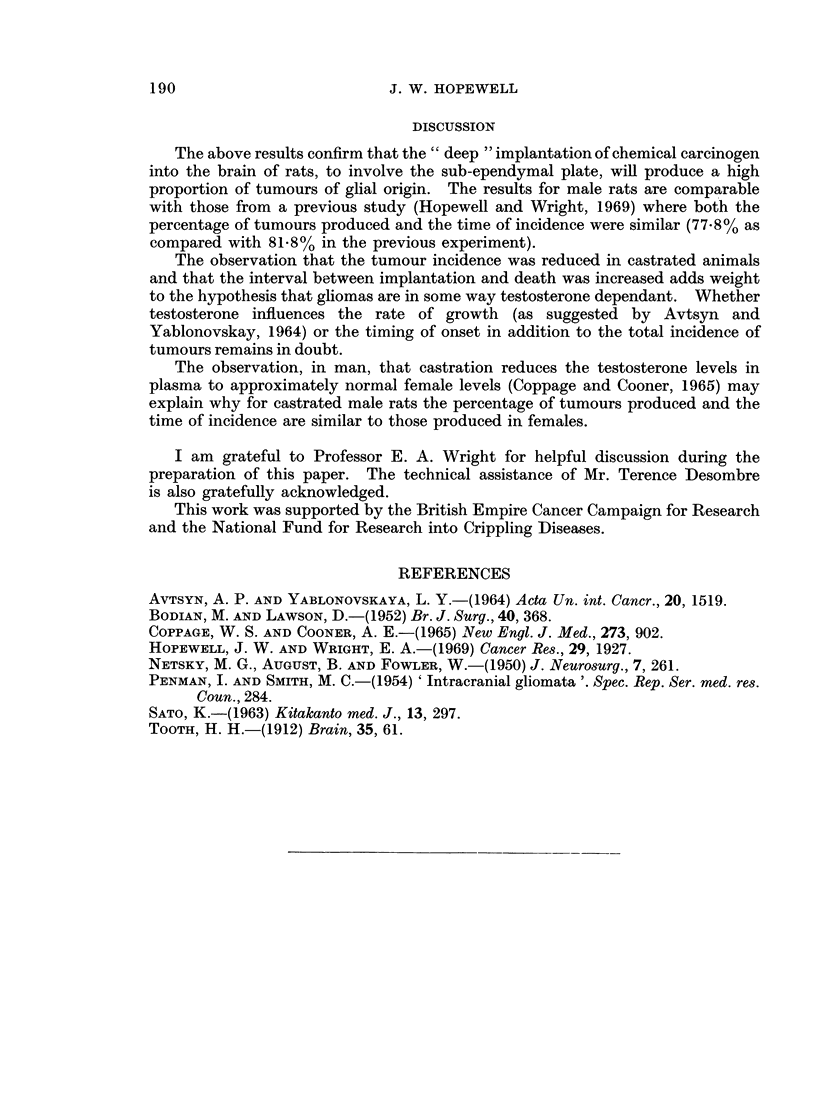# The Effects of Castration on the Induction of Experimental Gliomas in Male Rats

**DOI:** 10.1038/bjc.1970.21

**Published:** 1970-03

**Authors:** J. W. Hopewell

## Abstract

Pellets of 3,4-benzopyrene were implanted into the brain of equal numbers of normal and castrated male rats. The position of the implant was carefully controlled so that it impinged on the mitotically active sub-ependymal plate.

A high proportion of glial tumours (77.8%) were induced in normal male rats. The effects of castration were to reduce the incidence of tumoures (50%) and to increase the time interval between implantation and death from cerebral tumour.

The implications from these results, as to the possible roles of testosterone, are discussed.


### THE EFFECTS OF CASTRATION ON THE INDUCTION OF EXPERIMENTAL GLIOMAS IN MALE RATS

J. W. HOPEWELL

*From the *Department of Cellular Biology, St. Mary's Hospital Medical School, London, W.2*

Received for publication January 5, 1970

**SUMMARY.—Pellets of 3,4-benzopyrene were implanted into the brain of equal numbers of normal and castrated male rats. The position of the implant was carefully controlled so that it impinged on the mitotically active sub-ependymal plate.**

**A high proportion of glial tumours (77·8%) were induced in normal male rats. The effects of castration were to reduce the incidence of tumours (50%) and to increase the time interval between implantation and death from cerebral tumour.**

**The implications from these results, as to the possible roles of testosterone, are discussed.**

IT has been observed by several authors, both from a study of human and experimental material, that tumours of glial origin are more common in males (Bodian and Lawson, 1952; Hopewell and Wright, 1969; Netsky, August and Fowler, 1950; Penman and Smith, 1954; Sato, 1963; Tooth, 1912). Some of these authors have suggested that testosterone may be an important factor in the induction and growth of glial tumours (Avtsyn and Yablonovskaya, 1964; Hopewell and Wright, 1969; Penman and Smith, 1954).

A previous study (Hopewell and Wright, 1969) has shown that a high percentage of glial tumours can be produced in male rats by the deep implantation of a carcinogenic pellet into the brain so that it impinges on the mitotically active sub-ependymal plate. It seemed possible, using this method of tumour induction in normal and castrated male rats, that the hypothesis that testosterone is important in the induction and growth of glial tumours could be tested.

### MATERIALS AND METHODS

Under chloral hydrate anaesthesia (300 mg./kg. intraperitoneally) the brains of 20 4–6-week-old male Sprague Dawley rats received " deep " implantations of the carcinogen 3,4-benzopyrene so that the pellet involved the sub-ependymal plate of the lateral ventricle. The implantation was carried out as described previously (Hopewell and Wright, 1969).

At this time 10 of the rats were castrated by the surgical removal of the testes.

On recovery the rats were returned to the animal rooms where they were fed on Dixon's 41B diet and water *ad libitum*. Rats were observed at regular intervals

* Address from June 1, 1970: M.R.C. Radiobiology Unit, Churchill Hospital, Oxford.

until death or until they were killed *in extremis*. At the time of death the head was removed, the brain exposed and fixed by immersion in a solution of 1% acetic acid in 10% formol-saline. A more general post-mortem examination was also carried out to ascertain causes of death other than from cerebral tumour.

After fixation the brains were dissected out and sliced coronally. Histological sections were prepared in the normal way and stained with Ehrlich's haematoxylin and eosin.

At the time of death one castrated animal was found to have the pellet superficially situated in error and in the following evaluation of the result this animal has been excluded. The first rat in each experimental group died prematurely from a cerebral abscess before the time required to produce a cerebral tumour. These animals have also been excluded from the evaluation of the results.

Fig. 1.—Histograms to show the time and cause of death of all experimental animals.
T cerebral tumour
a lung infections
b cerebral abscess at site of operation
c head cannibalized
d slight internal hydrocephalus, cause unknown
sup. pellet superficially situated in error (animal died from lung infection).

### RESULTS

The histograms in Fig. 1 show the time and cause of death of all the experimental animals. It can be seen that following a " deep " intracerebral implant of 3,4-benzopyrene into normal and castrated male rats a higher proportion of glial tumours developed in normal males (7 out of 9; 77·8%) as compared with castrated rats (4 out of 8; 50%).

If the cumulative tumour incidence (expressed as a percentage of the total number of animals in the group) is plotted against time then it is also evident that tumours in normal males developed earlier than in castrated males. These results can be compared with previous results (Hopewell and Wright, 1969) which have been expressed in a similar way and super-imposed on Fig. 2.

The tumours produced were classified as gliomas, of various types, by their gross and histological appearance (Table I).

FIG. 2.—Cumulative number of cerebral tumours (expressed as a percentage of the number of animals) in control and castrated male rats plotted against the time between "deep" carcinogenic implant and death. The results from a previous experiment (Hopewell and Wright, 1969) for male and female rats have been expressed in a similar way and super-imposed for comparison.

●——● control males

○——○ castrated males

●......● males } Hopewell and Wright, 1969.

○......○ females }

TABLE I.—*Histological Types of Intracranial Tumours in this Investigation*

| Type | Number |
|---|---|
| Ependymoblastoma | 3 |
| Glioblastoma multiforme | 2 |
| Astrocytoma | 2 |
| Spongioblastoma | 2 |
| Oligodendroglioma | 1 |
| Mixed oligodendroglioma/astrocytoma | 1 |
| Total | 11 |

### DISCUSSION

The above results confirm that the " deep " implantation of chemical carcinogen into the brain of rats, to involve the sub-ependymal plate, will produce a high proportion of tumours of glial origin. The results for male rats are comparable with those from a previous study (Hopewell and Wright, 1969) where both the percentage of tumours produced and the time of incidence were similar (77·8% as compared with 81·8% in the previous experiment).

The observation that the tumour incidence was reduced in castrated animals and that the interval between implantation and death was increased adds weight to the hypothesis that gliomas are in some way testosterone dependant. Whether testosterone influences the rate of growth (as suggested by Avtsyn and Yablonovskay, 1964) or the timing of onset in addition to the total incidence of tumours remains in doubt.

The observation, in man, that castration reduces the testosterone levels in plasma to approximately normal female levels (Coppage and Cooner, 1965) may explain why for castrated male rats the percentage of tumours produced and the time of incidence are similar to those produced in females.

I am grateful to Professor E. A. Wright for helpful discussion during the preparation of this paper. The technical assistance of Mr. Terence Desombre is also gratefully acknowledged.

This work was supported by the British Empire Cancer Campaign for Research and the National Fund for Research into Crippling Diseases.

### REFERENCES

Avtsyn, A. P. and Yablonovskaya, L. Y.—(1964) *Acta Un. int. Cancr.*, **20**, 1519.
Bodian, M. and Lawson, D.—(1952) *Br. J. Surg.*, **40**, 368.
Coppage, W. S. and Cooner, A. E.—(1965) *New Engl. J. Med.*, **273**, 902.
Hopewell, J. W. and Wright, E. A.—(1969) *Cancer Res.*, **29**, 1927.
Netsky, M. G., August, B. and Fowler, W.—(1950) *J. Neurosurg.*, **7**, 261.
Penman, I. and Smith, M. C.—(1954) ' Intracranial gliomata '. *Spec. Rep. Ser. med. res. Coun.*, 284.
Sato, K.—(1963) *Kitakanto med. J.*, **13**, 297.
Tooth, H. H.—(1912) *Brain*, **35**, 61.